# Insights into the impact of manure on the environmental antibiotic residues and resistance pool

**DOI:** 10.3389/fmicb.2022.965132

**Published:** 2022-09-16

**Authors:** Luminita Gabriela Marutescu, Mihaela Jaga, Carmen Postolache, Florica Barbuceanu, Nicoleta Manuela Milita, Luminita Maria Romascu, Heike Schmitt, Ana Maria de Roda Husman, Paria Sefeedpari, Stefanie Glaeser, Peter Kämpfer, Patrick Boerlin, Edward Topp, Gratiela Gradisteanu Pircalabioru, Mariana Carmen Chifiriuc, Marcela Popa

**Affiliations:** ^1^Department of Microbiology, Faculty of Biology, University of Bucharest, Bucharest, Romania; ^2^Research Institute of University of Bucharest, Bucharest, Romania; ^3^The Institute for Diagnostic and Animal Health (IDSA), Bucharest, Romania; ^4^Faculty of Veterinary Medicine, University of Agronomic Sciences and Veterinary Medicine of Bucharest, Bucharest, Romania; ^5^National Institute for Public Health and the Environment, Bilthoven, Netherlands; ^6^Wageningen Livestock Research, Wageningen, Netherlands; ^7^Institute for Applied Microbiology Heinrich-Buff-Ring, Justus-Liebig University, Gießen, Germany; ^8^Department of Pathobiology, University of Guelph, Guelph, ON, Canada; ^9^Department of Biology, Agriculture and Agri-Food Canada, University of Western Ontario, London, ON, Canada; ^10^Academy of Romanian Scientists, Bucharest, Romania; ^11^The Romanian Academy, Bucharest, Romania

**Keywords:** antimicrobial resistance, antibiotic resistant bacteria, antibiotic residues, antibiotic resistance genes, manure, anaerobic digestion

## Abstract

The intensive use of antibiotics in the veterinary sector, linked to the application of manure-derived amendments in agriculture, translates into increased environmental levels of chemical residues, AR bacteria (ARB) and antibiotic resistance genes (ARG). The aim of this review was to evaluate the current evidence regarding the impact of animal farming and manure application on the antibiotic resistance pool in the environment. Several studies reported correlations between the prevalence of clinically relevant ARB and the amount and classes of antibiotics used in animal farming (high resistance rates being reported for medically important antibiotics such as penicillins, tetracyclines, sulfonamides and fluoroquinolones). However, the results are difficult to compare, due to the diversity of the used antimicrobials quantification techniques and to the different amounts and types of antibiotics, exhibiting various degradation times, given in animal feed in different countries. The soils fertilized with manure-derived products harbor a higher and chronic abundance of ARB, multiple ARG and an enriched associated mobilome, which is also sometimes seen in the crops grown on the amended soils. Different manure processing techniques have various efficiencies in the removal of antibiotic residues, ARB and ARGs, but there is only a small amount of data from commercial farms. The efficiency of sludge anaerobic digestion appears to be dependent on the microbial communities composition, the ARB/ARG and operating temperature (mesophilic vs. thermophilic conditions). Composting seems to reduce or eliminate most of antibiotics residues, enteric bacteria, ARB and different representative ARG in manure more rapidly and effectively than lagoon storage. Our review highlights that despite the body of research accumulated in the last years, there are still important knowledge gaps regarding the contribution of manure to the AMR emergence, accumulation, spread and risk of human exposure in countries with high clinical resistance rates. Land microbiome before and after manure application, efficiency of different manure treatment techniques in decreasing the AMR levels in the natural environments and along the food chain must be investigated in depth, covering different geographical regions and countries and using harmonized methodologies. The support of stakeholders is required for the development of specific best practices for prudent – cautious use of antibiotics on farm animals. The use of human reserve antibiotics in veterinary medicine and of unprescribed animal antimicrobials should be stopped and the use of antibiotics on farms must be limited. This integrated approach is needed to determine the optimal conditions for the removal of antibiotic residues, ARB and ARG, to formulate specific recommendations for livestock manure treatment, storage and handling procedures and to translate them into practical on-farm management decisions, to ultimately prevent exposure of human population.

## Introduction

Antimicrobial resistance (AMR) is ranked in the top 10 public health global challenges and top 20 global causes of mortality and morbidity according to WHO projections for 2030, causing a real health care crisis, worsened by the innovation gap in the development of novel antimicrobial drugs [World Health Organization (WHO), 2015, 2021]. Even if clinical use is the main driver for AMR increase in humans, the environment represents both an important AMR reservoir (natural or intrinsic resistance being an essential component of the armamentarium used by different microbial species to maximize competition, resist predation and colonize different niches) and an important transmission route to humans (Walsh, 2013; Nguyen et al., 2020).

**Figure 1:**
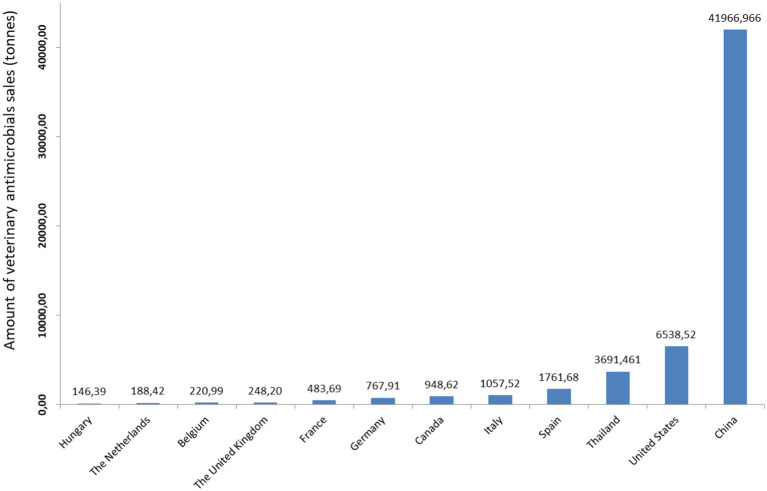
Reported sales of veterinary antimicrobials by country in 2017 (Tiseo et al., 2020). The figure is created using Biorender.com.

The intensive use of antibiotics in the clinical, industrial, veterinary and agricultural sectors led to increased environmental levels of chemical residues, AR bacteria (ARB) and antibiotic resistance genes (ARG). It was estimated that the global antimicrobials consumed in livestock production was 93,309 tonnes in 2017, and projected an increase of 11.5% by 2,030–104,079 tonnes (95% CI: [69,062, 172,711]) (Boeckel et al., 2017; Tiseo et al., 2020; Figure 1). The joint Food and Agriculture Organization of the United Nations (FAO), WHO and World Organization for Animal Health (OIE) expert meeting document on foodborne AMR shows that >80% of antimicrobials and dietary copper and zinc is excreted in active form (FAO and WHO, 2019) and accumulate in different environmental compartments.

Under the selection pressure of different chemical contaminants released and accumulated in different environmental compartments, ARG can be exchanged through transduction or conjugation, between even distantly related bacteria (van Hoek et al., 2011; Vikesland et al., 2017; Popa et al., 2018) or novel resistance factors could occur through mutation (Huijbers et al., 2019). Sub-inhibitory levels of selective agents such as antibiotics or heavy metals have been shown to increase the abundance of ARG and mobile genetic elements (MGEs) through co-resistance (selectable for different ARG on the same MGE) and cross-resistance (selectable for an ARG encoding for a common mechanism of resistance to both antibiotics and biocides; Jutkina et al., 2016; Zhang et al., 2018; Cao et al., 2020). Moreover, the ARG associated with dead bacteria or free DNA could also contribute to acquired resistance through transformation (Woegerbauer et al., 2020). Also, the environmental pollution with antibiotic residues may alter the soil microbiota composition, enriching soils with bacteria able to derive nutritional benefits from these drugs, resulting in their accelerated biodegradation (Topp et al., 2013).

The increase in manure inputs and/or agriculture derived antibiotics due to the intensification of livestock production raises serious concerns for both human and environmental health, following direct application of manures to farmlands (e.g., exposure of crops and consumers to ARB, emergence/accumulation/spread of resistance to antibiotics used human medicine etc.; Van Epps and Blaney, 2016; Wepking et al., 2017; Collignon and McEwen, 2019).

The aim of this review is to provide insights into the impact of animal farming and manure application on antibiotics residues and antibiotic resistance in the environment, to identify the knowledge gaps and highlight the best practices for livestock manure treatment, storage and handling procedures and for prudent-cautious use of antibiotics in livestock production.

## Livestock production as a source of antibiotic residues

**Figure 2:**
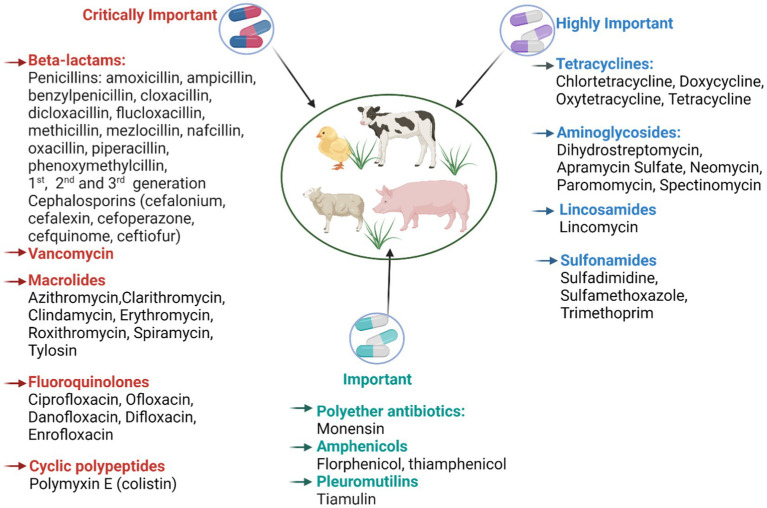
Major classes of antibiotics and representatives used in livestock production. The figure is created using Biorender.com.

One of the critical points to mitigate dissemination, accumulation and transmission of AMR into the environment is to identify the reservoirs of ARB, ARG and antibiotics. Worldwide, it is estimated that 73% of all antibiotics are used in livestock farming, not in human medicine, supporting a growing awareness of the associated risks (Boeckel et al., 2017; Van et al., 2020). A large proportion of these antimicrobials are used for controlling infectious diseases that would otherwise inflict severe losses or even prevent intensive production completely (Aarestrup, 2015). WHO reports that 57% of all antimicrobials used in animal production are essential for human medicine (Figure 2). In this regard, CIA (critically important antimicrobials) lists have been created by WHO and national governments in order to prioritize and protect the most essential antibiotics [World Health Organization (WHO), 2019].

The FDA has highlighted three CIA classes, i.e., third generation cephalosporins, macrolides and fluoroquinolones as the critically important classes. According to the most recent FDA (2020) report on antibiotic sales, these three CIA classes accounted for around 5% of total antibiotics sales for use in farm animals (FDA, 2020). The same three, along with fourth generation cephalosporins, were tagged by WHO as priorities for attention and funding in the battle against AMR.

In the US, 70%–80% of the total antibiotics sold per year are used in animals (Elliott et al., 2017) and according to the FDA, 57% of medically important ones belong to the same classes as drugs used in human health (FDA, 2020). In the US, 82% of the medically important antibiotics were estimated to be used in cattle (41%) and swine (941%; FDA, 2020). Tetracyclines are among the most widely used in the US, accounting for 66%–67% of the medically important antimicrobials, being primarily sold for use in cattle and swine (FDA, 2019, 2020; Scott et al., 2019).

According to the fourth edition of OIE annual report on antimicrobial agents intended for use in animals, in Europe, bovines account for the highest percentage of antibiotic consumption (38.3%) followed by swine (27.1%; OIE, 2018). The total use of antibiotics in the EU has been estimated in different studies to range from 20 to 188 mg kg^−1^ of animal (slaughtered pigs, poultry and cattle plus estimated biomass of live dairy cattle; Grave et al., 2010; Pikkemaat et al., 2016). In the EU, tetracyclines accounted for <40% (35.3%) in 2016 (OIE, 2018).

Antibiotics administered to animals modify the gut microbiota signatures, as demonstrated for chlortetracycline, sulfamethazine, and penicillin which induced, after 14 days of administration in swine, an increase in *Escherichia coli* populations, known for its pathogenic potential, but also in the abundance and diversity of ARGs, some of them, such as aminoglycoside O-phosphotransferases, conferring resistance to antibiotics that were not administered, demonstrating the potential for indirect selection of resistance to classes of antibiotics not fed (Looft et al., 2011). Moreover, the decrease of gut diversity by antibiotics might facilitate the overgrowth of already resistant microbes and the HGT of ARGs. It has been demonstrated that antibiotics could promote the HGT by: (i) activation of the SOS response, which increases the transfer of diverse mobile genetic elements (pathogenicity islands, integrating conjugative element, conjugative transposons, plasmids etc.; Couce and Blázquez, 2009); (ii) promoting transduction, by stimulating prophage excision and host cell lysis and also the receptor strain (Hastings et al., 2004; Lopatkin et al., 2016); (iii) increasing PT, as demonstrated, e.g., for ESBL carrying plasmids in *E. coli* (Liu et al., 2019). Moreover, the mutations and recombinations are also increased in the presence of antibiotic stress (Couce and Blázquez, 2009).

Antibiotics released in the natural environment could affect microbial diversity with important ecological consequences (Duygan et al., 2021).

The use of antibiotics in livestock production is mirrored by the presence of these agents in animal manures. Animal manures are mixtures of animal feces, urine, bedding materials and other materials associated with animal production (Shober and Maguire, 2018). Due to their high organic nutrient content of animal manures, they are widely used as natural fertilizers to increase food production yields. The antibiotic residue concentrations found in manures vary from study to study (Table 1). There are at least three reasons for this occurrence: (i) the diversity of the quantification techniques, recovery efficiencies, sensitivity and reliability measures; (ii) having different quantities and types of antibiotics, with different degradation times (DT) given in animal feed which differ from country to country; (iii) different fate of antibiotics in the animal gut (catabolization pathway, the resulting residues) and soil (biodegradation depends on the structure of soil microbiota etc.). In relation to the first reason, suitable analytical methods for the quantification of antibiotics in the complex sample matrix represented by animal manure and treatment products are needed. A single liquid–liquid extraction step and analysis *via* liquid chromatography (LC) and triple quadrupole mass spectrometry, with a detection limit ranging from 0.01 to 0.08 mg/kg has been developed for the analysis of different sulfonamides and tetracyclines in biogas plant input and output samples (Spielmeyer et al., 2014). Antibiotics can be removed by physical adsorption, chemical oxidation, photodegradation, and biodegradation (Liu et al., 2021). Although biodegradation is receiving increasing attention due to its multiple advantages (green, low cost and easy to perform operation), it must be taken into account that many of the clinically important antibiotics are not easily biodegradable (Alexy et al., 2004).

Fluoroquinolone, sulfonamide, and tetracycline classes are the most widely detected antibiotics in different types of manure (Table 1). One study has shown that the consumption of tetracyclines, as the most frequently detected antibiotics in agricultural soils fertilized with animal manure, was directly correlated with the size of size of the farm and the amounts of tetracyclines found in the fertilized soils (Carballo et al., 2016).

As shown in Table 1, the amount of antibiotics in swine, poultry and cattle manure ranged between 0.01 and 100 mg/kg, the concentration can be higher, of >1,000 mg/kg, however (Youngquist et al., 2016).

**Table 1:** The amount of antibiotics detected in poultry, swine and cattle manure in different countries.

Animal manure	Classes of antibiotics (antibiotics)	Concentration (mg/kg)	Country	References
Poultry	Quinolones (Enrofloxacin)	0–1,421 0–31 0–8	China Egypt Austria	Zhao et al. (2010) Leal et al. (2012) Martínez-Carballo et al. (2007)
	Sulfonamides	0–6	Austria	Martínez-Carballo et al. (2007)
	Sulfonamides (Sulfadiazine)	0-51	China	Zhao et al. (2010)
	Tetracyclines (Chlortetracycline)	0-66	US	Furtula et al. (2010)
	Diaminopyrimidines (Trimethoprim)	0–17	Austria	Martínez-Carballo et al. (2007)
Swine	Quinolones (Enrofloxacin)	0.006–0.033	Belgium	Rasschaert et al. (2020)
	Sulfonamides	0–100 0–0.2 0.1–0.23 0.2–1.0	US China Switzerland Germany	Campagnolo et al. (2002) Zhao et al. (2010) Haller et al. (2002) Hamscher et al. (2005)
	Sulfonamides (Sulfadiazine)	0.02-3	Belgium	van den Meersche et al. (2016)
	Polymyxins (Colistin)	0.6–48.6	Belgium	van den Meersche et al. (2016)
	Tetracyclines	0.6–66 0.009–0.025 0.042–0.698	Germany Belgium Denmark	Harms and Bauer (2012) Rasschaert et al. (2020) Harms and Bauer (2012)
	Tetracyclines (Oxytetracycline)	0.011–3.8	Belgium, Spain Italy	Carballo et al. (2016), van den Meersche et al. (2016), and Rasschaert et al. (2020)
	Tetracyclines (Chlortetracycline)	0.013-0.058 0.56	Belgium Spain	Rasschaert et al. (2020) Carballo et al. (2016)
	Tetracyclines (Doxycycline)	0.4 – 22	Belgium Netherlands Spain	Berendsen et al. (2015), Carballo et al. (2016), van den Meersche et al. (2016), and Rasschaert et al. (2020)
Cattle	Quinolones (Danofloxacin, Difloxacin Enrofloxacin Ciprofloxacin)	0.4–46	China	Zhao et al. (2010) and Li et al. (2013)
	Sulfonamides	0–0.4 0–10	US China	Watanabe et al. (2010) and Wallace and Aga (2016) Zhao et al. (2010) and Li et al. (2013)
	Tetracyclines	0–1.2 0.4–27	US China	Watanabe et al. (2010) and Wallace and Aga (2016) Storteboom et al. (2007) and Li et al. (2013)
	Tetracyclines (Oxytetracycline)	0-20 0–0.5 0.210–103	Italy US China	De Liguoro et al. (2003) Watanabe et al. (2010) and Wallace and Aga (2016) Storteboom et al. (2007) and Zhao et al. (2010)
	Tetracyclines (Chlortetracycline)	0-0.1 0.02–0.5 0.2–27	US Germany, Spain China	Watanabe et al. (2010) and Wallace and Aga (2016) Storteboom et al. (2007), Kemper (2008), and Carballo et al. (2013) Zhao et al. (2010) and Li et al. (2013)
	Tetracyclines (Doxycycline)	0-0.02 0.4–10	Germany, Spain China	Kemper (2008) and Carballo et al. (2013) Zhao et al. (2010) and Li et al. (2013)

**Figure 3:**
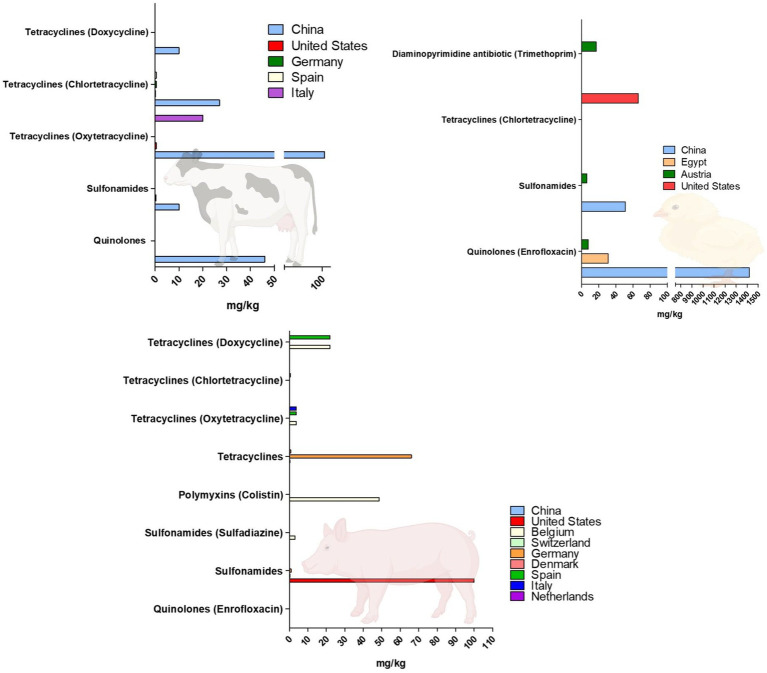
Graphic representation of the highest concentrations of antibiotics detected in animal manure (cattle, poultry, swine), reported in different studies. The figure is created using Biorender.com.

The highest concentrations are reported in poultry, for fluoroquinolones, in cattle for oxytetracycline and for sulfonamides in swine manure (Figure 3).

Therefore, antibiotic residues enter the environment through the use of animal wastes, raising increasing concerns about their contribution to the AMR reservoir (Amador et al., 2019; Tyrrell et al., 2019).

Some of the antibiotic residues are excreted intact in manure, where they form complexes with soluble organics, preventing their degradation and removal during manure storage (Massé et al., 2014). Other antibiotics such as sulfamethazine, tylosin and chlortetracycline are rapidly degraded in manure amended soils, the half-live or DT_50_ ranging from 2 to 42 days (Halling-Sørensen et al., 2005; Carlson and Mabury, 2006; Accinelli et al., 2007; Topp et al., 2013). The half-life of oxytetracycline in manure from calves was 30 days but the compound was still detectable in this matrix (820 μg/kg) after 5 months maturation (De Liguoro et al., 2003).

## Animal manure as a reservoir of ARB and ARG

**Figure 4:**
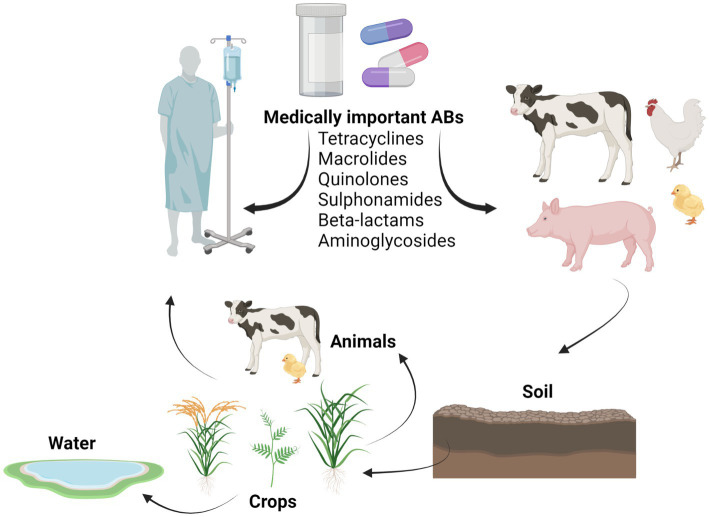
Contribution of manure to the flux of antibiotics from livestock to the natural environment. The figure is created using Biorender.com.

Currently, the application of manure and slurry in agriculture is considered as a key contributor in the flow of ARG among humans, animals and terrestrial and aquatic environments (Figure 4; Topp et al., 2018; Durso and Cook, 2019). Many studies have shown that the application of manure from antibiotic-treated animals to soil was found to enlarge the reservoir of clinically relevant ARB and ARG (Table 2) when compared to soils that received inorganic fertilizers or no fertilizers (Storteboom et al., 2010; Heuer et al., 2011; Cao et al., 2020).

**Table 2:** ARB found in manure.

**Animal manure**	**Bacteria**	**Antibiotic resistance patterns**	**Resistance phenotype (%)**	**Location**	**References**
Poultry manure	*Enterobacteriaceae*	TET, SXT, CHL, AMC	MDR (71%)	Portugal	Amador et al. (2019)
Pig manure	*Enterobacteriaceae*	TET, SXT, CHL, AMC, ATM, CTX	MDR (79%)	Portugal	Amador et al. (2019)
Cattle manure	*Enterobacteriaceae*	AMC, TET, CHL, SXT MEM	MDR (69%)	Portugal	Amador et al. (2019)
Pig manure	*E. coli*	CHL, SXT, DOX, S, AK, CS, IMP, NN	ESBL (1.6%) MDR (52.2)	Germany	Hölzel et al. (2010)
Pig manure	*Enterococcus faecalis*	DOX, ERY, RIF, IMP, LZD	MDR (76.2%)	Germany	Hölzel et al. (2010)
Pig manure	*E. faecium*	RIF, ERY, DA, DOX, EFX, FOS, MOX, S	MDR (87.9%)	Germany	Hölzel et al. (2010)
Mixed manure of livestock husbandry	*E. coli*		ESBL	Germany	Schauss et al. (2015)
Pig manure	*Salmonella*	S, SXZ, TET, CTF, CRO, FOX	MDR (58.73%)	US	Pornsukarom and Thakur (2016)
Poultry manure	*E. faecium*	VAN, TET, SXT, CIP, ERY, BA	VRE (14.4%)	Greece	Tzavaras et al. (2012)
Poultry manure	*E. faecium*	QD, ERY, CIP, DA, GEN		US	Graham et al. (2009)
Poultry manure	*E. faecalis*	DA, GEN, ERY, TET		US	Graham et al. (2009)
Poultry manure	*Staphylococcus*	ERY, SG, TET		US	Graham et al. (2009)
Chicken and pig manure	*E. coli*	AMC, CTX, GEN, KAN, TE, CIP, ERY, SXT, CHL, VAN		China	Yang et al. (2017)
Cattle manure	*E. coli*	AMP, AZT, CTX, CAZ, CHL, CIP, GEN, NA, SXT, TET, TMP		Belgium	Huygens et al. (2021)
Faecal samples of broilers, pigs, dairy cows, calves	*E. coli*	AMP, AMC, CTX, CRO, TET, SXT, TMP, CHL, GEN	ESBL/AmpC (32.6% in broilers) ESBL/AmpC (32.6% in veal calves) ESBL/AmpC (10% in pig) MDR (31.4 in broilers; 27.3%) in pigs; 27% in calves, 2.1% in dairy cattle	Netherlands	MARAN (2018)
Fecal specimen of broiler pigs and dairy cattle	*E. coli*		ESBL	Germany	Friese et al. (2013)

TET, tetracycline; DOX, doxycycline; S, sulphonamides; TMP, trimethoprim; SXT, cotrimoxazole; CHL, chloramphenicol; AMC, amoxicillin + clavulanic acid; ATM, aztreonam; MEM, meropenem; IMP, imipenem; CTX, cefotaxime; CTF, ceftiofur; CRO, ceftriaxone; FOX, cefoxitin; AK, amikacin; NN, tobramycin; GEN, gentamicin; KAN, kanamycin; CS, colistin sulfate; ERY, erythromycin; DA, clindamycin; RIF, rifampicin; LZD, linezolid; FOS, fosfomycin; NA, nalidixic acid; EFX, enrofloxacin; MOX, moxifloxacin; CIP, ciprofloxacin; VAN, vancomycin; BA, boronic acid; QD, quinupristin-dalfopristin; SG, streptogramin.

Several studies reported correlations between the prevalence of clinically relevant ARB and the amount and classes of antibiotics used in livestock production (Landers et al., 2012; Chantziaras et al., 2013; EMA, 2019). In the Netherlands, levels ranging from 10*^2^ to 10*^4^ CFU/g of *E. coli*, spores of sulfite reducing Clostridia and intestinal enterococci have been found in the input stream and were correlated with the presence of these microorganisms in the RO-concentrate of nine installations producing mineral concentrate from pig slurry (Hoeksma et al., 2015, 2020, 2021).

However, most studies investigating the presence of ARB in livestock have focused on *Enterobacteriaceae* including *Escherichia coli* and zoonotic *Salmonella* spp. (Feßler and Schwarz, 2017) and fewer have focused on other genera, such as *Enterococcus, Staphylococcus* etc. (Oliver et al., 2020). The available reports indicated high resistance rates for the most frequently used antibiotic classes: beta-lactams (Dandachi et al., 2018), tetracyclines and sulfonamides (Amador et al., 2019). For some of the nine European countries included in a cross-sectional study, an association was observed between the use of penicillins and resistance to ampicillin in isolates from healthy broilers (Hou et al., 2017; Ceccarelli et al., 2020). The *E. coli* isolates from broilers exhibited the highest resistance to (fluoro) quinolones, and multidrug resistance (MDR) was also detected in broilers and fattening turkeys (Ceccarelli et al., 2020). The decrease of colistin usage in the veterinary medicine world widely led to the decrease of colistin resistance to very low levels (Doi, 2019; Miguela-Villoldo et al., 2022). In the Netherlands, a considerably reduced antimicrobial consumption in food-producing animals was correlated with a low prevalence of ARB from broilers (MARAN, 2018). Similarly, in Germany, the level of AMR in commensal *E. coli* from livestock has been significantly decreased (EFSA, 2018).

Many studies have indicated that farmlands could host multiple ARG (Table 3; Baquero et al., 2008; Cheng et al., 2013; Udikovic-Kolic et al., 2014; Wichmann et al., 2014; Rahman et al., 2018). Moreover, Graham et al. (2016) suggested a historical association between ARG in animal manure and humans, demonstrating that higher β-lactam ARG levels were detected in soils fertilized with manure versus those amended with inorganic fertilizers, after the introduction of penicillin in 1940 (Graham et al., 2016). A cross sectional study regarding possible correlations between antimicrobial usage and the pig faecal mobile resistome conducted in nine European countries provided robust evidence of direct ARG selection by the two widely used antimicrobial classes, i.e., macrolides and tetracyclines (Van Gompel et al., 2019). Additionally, cross-resistance to macrolide and lincosamide usage and co-selection of ARG was also observed (Van Gompel et al., 2019). In a very recent study conducted in a high-density farming area of Northern Italy, it has been found that flumequine concentrations increased after manure application, positively correlated with the *oqxA* and *qnrS* genes abundance (Laconi et al., 2021). A recent metagenomic study evaluated the impact of bovine and poultry manure on the diversity and abundance of ARGs and the associated mobilome in soil and crops (Buta-Hubeny et al., 2022). The poultry manure contributed four times more than bovine manure to the total number of ARGs for different classes of antibiotics (tetracyclines, aminoglycosides, sulfonamides, bacitracin, chloramphenicol, and macrolide-lincosamide-streptogramin) found in manure, the fertilized soil and bacteria isolated from crops (Buta-Hubeny et al., 2022).

**Table 3:** The ARGs detected in animal manure.

**Animal manure**	**ARG in manure amended soil**	**References**
Pig and chicken	*aad*	Pu et al. (2019)
Pig and chicken	*aphA3*	Pu et al. (2019)
Dairy	*bla*_CTX-M_	Marti et al. (2013), Hu H. et al. (2016a), Hu Y. et al. (2016b), and Nõlvak et al. (2016)
Dairy and pig	*ermB*	Marti et al. (2013), Marti et al. (2014), Hu H. et al. (2016a), Hu Y. et al. (2016b), Pu et al. (2019), Van den Meersche et al. (2019), and Van den Meersche et al. (2020)
Pig	*ermF*	Van den Meersche et al. (2020)
Dairy	*sul*1	Munir and Xagoraraki (2011), Marti et al. (2013), Pruden et al. (2013), Fahrenfeld et al. (2014), Ross and Topp (2015), Sun et al. (2015), Nõlvak et al. (2016), Ruuskanen et al. (2016), and Woegerbauer et al. (2020)
Pig and chicken	*sul*2	Selvam et al. (2012), Pu et al. (2019), and Van den Meersche et al. (2019)
Dairy	*tet(A)*	Marti et al. (2013), Hu H. et al. (2016a), Hu Y. et al. (2016b), Nõlvak et al. (2016), Sandberg and LaPara (2016), and Yuan et al. (2020)
Dairy and pig	*tet(W)*	Selvam et al. (2012), Kyselková et al. (2013), Fahrenfeld et al. (2014), Kyselková et al. (2015), Sandberg and LaPara (2016), Pu et al. (2019), and Woegerbauer et al. (2020)
Dairy cattle and swine	*tet(M)*	Sun et al. (2015) and Van den Meersche et al. (2020)
Dairy manure	*tet(X)*	Sandberg and LaPara (2016) and Woegerbauer et al. (2020)
Pig and chicken	*tet(L)*	Pu et al. (2019)
Pig and chicken	*tet(O)*	Pu et al. (2019)
Pig and chicken	*tet(Q)*	Selvam et al. (2012), Pu et al. (2019), and Van den Meersche et al. (2019)
Dairy	*intI*1	Marti et al. (2013), Marti et al. (2014), Kyselková et al. (2015), Hu H. et al. (2016a), Hu Y. et al. (2016b), Nõlvak et al. (2016), and Woegerbauer et al. (2020)
Pig and chicken	*mexF, vgb, vanSC*	Pu et al. (2019)
Dairy cattle and swine farms	*blaOXA-58*	Ruuskanen et al. (2016)
Cattle, swine, poultry, and pork	*OXA-type β-lactamases*	Li et al. (2015)
Cattle	*ampC*	Wepking et al. (2017)
Cattle	*β-lactam-resistance genes (blaCEP-04 gene)*	Fang et al. (2015)
Pig	*gyrA, parC*	Selvam et al. (2012)
Pig	*qnrS, tetW, ermB, sul1, bla_KPC_,*	Gros et al. (2019)
Dairy cattle, chickens and swine manure	*ermA, ermB, blaOXA-1, oqxA*	Laconi et al. (2021)

Antibiotic resistance genes can persist in soil for >120 days (Gou et al., 2018; Han et al., 2018) and can take from three to 6 months to attenuate to levels less than or equal to background (Chen et al., 2019; Lopatto et al., 2019). This implies that soils which are in constant agricultural use might continuously harbor a relatively stable ARB population and ARG pool (Schmitt et al., 2006; Cheng et al., 2013; Ruuskanen et al., 2016). However, more evidence, including quantitative data from exposure-relevant sites and environmental compartments is needed to evaluate the contribution of antibiotics containing manures to the environmental selection for AMR and its dispersal routes (Huijbers et al., 2015; Hassell et al., 2019). This evidence will allow the implementation of targeted monitoring programs and interventions to prevent resistant pathogens, as well as novel ARG, from reaching humans (Bengtsson-Palme et al., 2018; MARAN, 2018).

Studies are also reporting evidence for the potential of ARG transfer between animal-related and human-associated bacteria (Hu H. et al., 2016a, Hu Y. et al., 2016b; Pal et al., 2016; Pérez-Valera et al., 2019; Yuan et al., 2020). Manure offers some particular features, such as nutrients richness, high abundance and diversity of bacterial populations and antibiotic residues, that could favor the ARGs dissemination by horizontal gene transfer (Heuer et al., 2011; Lima et al., 2020). The insertion sequences (IS), including those associated with the mobility of ARGs in the population of ESKAPE pathogens, are introduced to soil with manure and remain stable for up to several months, indicating an increased risk of rapid ARG transfer, particularly when associated with bacteria from phylum *Proteobacteria* (Buta-Hubeny et al., 2022).

The abundance of 95 ARGs and MGEs from soils fertilized with dairy cow manure-derived amendments (slurry, fresh manure, aged manure), and plants (wheat grain and lettuce) was analyzed by high-throughput qPCR. The structure of soil prokaryotic communities was determined by 16S rRNA amplicon sequencing and qPCR (Jauregi et al., 2021). A higher ARGs abundance was found in slurry vs. fresh or aged manure, in soil vs. plant samples, and in wheat grain vs. lettuce (Jauregi et al., 2021; Zalewska et al., 2021).

## Manure treatment impacts on persistence, selection, enrichment and dissemination of AMR

**Figure 5:**
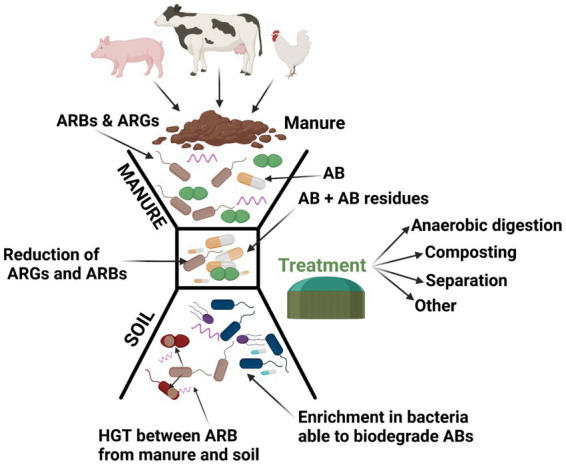
Effects of manure treatment on ABs and ARB, ARGs. The figure is created using Biorender.com.

The available manure processing technologies are currently not taking into consideration, in terms of treatment performance, the removal of antibiotics, ARG and ARB, although evidence exists that ARB and ARG are not completely eliminated during manure treatments, contributing to the environmental resistome (Hou et al., 2017; Figure 5).

In a recent review, it has been shown that the uncovered lagoons are the least effective removing tetracycline resistance gene; however, their performance could be gradually improved if covered lagoons and biofiltration are sequentially added. Further, mesophilic digestion was not effective, in contrast with thermophilic AD. Aerobic thermophilic composting and postdigestion composting reduced ARGs by >80% (Agga et al., 2022). These results suggest that different manure management practices have different efficiencies in removing antibiotics, ARB and ARG. In the following sections of the manuscript, we will focus on the efficiency of anaerobic digestion (AD) and composting techniques.

Liquid (with a dry matter content of 6%–12% or less if flushing is applied) or solid (with a dry matter content of 20%–65%) animal manure are treated by different technologies to obtain organic fertilizer products. In the first step, the liquid manure is separated into two fractions, i.e., the liquid (3%–6% dried matter content), which is generally applied as fertilizer and the solid one.

The tetracycline residues are largely removed from the liquid fraction, the antibiotic being absorbed onto the solid fraction of manure. Thus reduction of the antibiotic load in the liquid fraction is an important first step in manure treatment (Wallace et al., 2018). On the contrary, to their poor absorption in the animal gut and/or reversible metabolization, sulfonamides are excreted in significant amounts and are persisting in the liquid manure and then entering the environment. In the groundwater samples from Lower Saxony agricultural areas, sulfonamides were detected in concentrations up to 100 ng L^−1^ (Spielmeyer et al., 2017).

The solid fraction can be further composted, dried and eventually pelletized or incinerated, while the liquid fraction can be used as fertilizer (eventually contributing to the dissemination of antibiotic residues, ARG and zoonotic bacteria into the environment) or further treated by different procedures (Lyngsø et al., 2011; Dungan et al., 2018; Van den Meersche et al., 2019; Oliver et al., 2020).

Both liquid and solid manure can be used for biogas production in AD systems, which are the most frequent treatments in Europe, being applied to treat 6.4% of the total manure production in 2010 (LIFE+MANEV, 2015).

Another common treatment of manure is represented by composting (microbial decomposition of organic matter under controlled aerobic conditions), the resulting product being more suitable for direct soil application. The aerobic composting process begins with the activity of mesophilic organisms, at 20–45°C, which will generate the appropriate conditions for the activity of thermophilic fungi and bacteria, with optimum growth temperature of 50–70°C. Regular turning of the pile will speed up the decomposition process, from several months in case of extensive outdoor composting in windrows with limited turning to 1–2 weeks in intensive tunnel composting with forced aeration (Melse and de Buisonjé, 2020).

### Effect of AD and composting on antibiotic residues

#### Anaerobic digestion

The rates of degradation of antibiotics during AD are dependent on their chemical structure, but also on manure characteristics and type, the reported degradation rates differing from study to study (Arikan et al., 2009; Alvarez et al., 2010; Kasumba et al., 2020; Lee et al., 2020).

**Figure 6:**
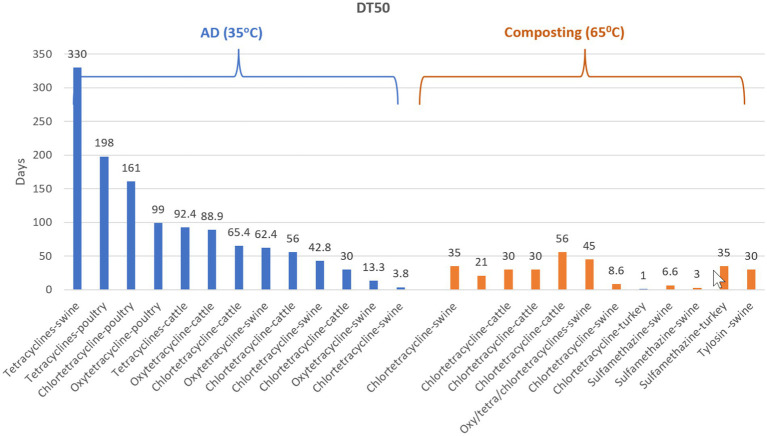
Comparative representation of the DT50 of different antibiotics during AD and composting of different types of manure (poultry, swine, cattle, turkey), reported in different studies (Osman et al., 2006; Dolliver et al., 2008; Alvarez et al., 2010; Hu et al., 2011; Kim et al., 2012; Selvam et al., 2012; Ray et al., 2017; Kasumba et al., 2020; Lee et al., 2020). The figure is created using Biorender.com.

Tetracycline and sulfonamide antibiotics were shown to persist in the animal manure (swine, cattle, and poultry) after AD, which can potentially lead to the emergence and persistence of tetracycline resistant bacteria in the environment when the AD byproducts are applied on soils for crop production (Spielmeyer et al., 2014, 2015, 2017; Kasumba et al., 2020). In case of tetracyclines, the DT50 during AD reported in different studies varied from >300 to 3.8 days in comparison with composting (Figure 6).

#### Composting

Composting reduces or eliminates many antibiotic residues in manure mainly by temperature dependent abiotic processes such as adsorption and/or degradation rather than biotic processes (Dolliver et al., 2008; Arikan et al., 2009; Wu et al., 2011; Kim et al., 2012; Ray et al., 2017; Liu et al., 2018; Cheng et al., 2019). For most antibiotics the calculated half-lives during composting ranged from 0.9 to 16 days (Oliver et al., 2020; Figure 6). In the case of cow manure stockpiles, the degradation of antibiotics takes place in weeks, months or longer and is dependent on the type of antibiotics, and interaction between different antibiotics (Oliver et al., 2020). For example, chlortetracycline was found to decline rapidly, with <1% of the starting concentration detectable after day 17, in stockpiled cattle manure. However, the degradation rate slowed down to 56 days when chlortetracycline and sulfamethazine were both present in a stockpile (Sura et al., 2014). These results suggest that when multiple antibiotics are present, some of them could inhibit the bacteria able to degrade other antibiotics (Oliver et al., 2020). However, in case of other antibiotics associations, the degradation rates are enhanced probably due to the selection of compost microbial associations able to act on multiple compounds (Storteboom et al., 2007; Arikan et al., 2009).

### Effects of AD and composting on ARB and ARG

#### Anaerobic digestions

The efficiency of AD in the removal of ARB and ARG appears to be dependent on the digester operating temperature. Anaerobic digestion at thermophilic conditions seems to be better than mesophilic AD at reducing ARB and ARG (Sun et al., 2016; Wallace et al., 2018; Gros et al., 2019; Huang et al., 2019; Zou et al., 2020). Zou et al. (2020) found that during thermophilic AD of pig manure, ARB numbers (sulfonamide / tetracycline resistant bacteria) were reduced by 4-log CFUs per gram dry manure, but only by approximately 1-log CFU at mesophilic temperature. Sun et al. (2016) observed that tetracycline resistance genes decreased when the operating temperature was in the thermophilic range. Eight out of 10 detected ARG declined at 55°C, but only five out of 10 and four out of 10 ARG decreased at 35°C and 20°C, respectively (Sun et al., 2016). However, Huang et al. (2019) found that thermophilic AD of swine manure did not achieve a better removal of abundance of total ARG compared to mesophilic AD.

Mesophilic AD seems to work only for certain ARB/ARG. For example, Wallace et al. (2018) noticed a significant reduction in sulfonamide resistance genes during mesophilic AD of cow manure, but no reduction effect on tetracycline resistance genes. Moreover, some other studies showed even an increase of the abundance of some ARG after mesophilic AD treatment such as sulfonamide, amphenicol and tetracycline resistance genes (Chen et al., 2010; Pu et al., 2018). Gros et al. (2019) observed that the mesophilic AD treatment, even though it was followed by solid–liquid separation, has reduced only modestly the abundance of ARG for quinolones, tetracyclines, macrolides, sulfonamide and β-lactams, as the copy numbers detected in the solid and liquid digestate fractions were similar to those quantified in slurry and sludge (Gros et al., 2019).

The different efficiency in removing ARG between mesophilic and thermophilic AD was linked to the microbial community composition of the sludge AD process (Agga et al., 2020). The microbial communities may decrease the antibiotic concentration during the mesophilic AD process, the subinhibitory antibiotic concentrations potentially acting as selection factors and promoting the propagation of ARB that could explain the increased abundance of ARG (Agga et al., 2020).

However, further studies for better understanding the ARG evolution under different setups are needed.

Although lab-scale AD has been demonstrated to reduce the abundance of ARG, there are only few data from commercial farms. The impact of on-farm AD on the decrease of enteric bacteria ARG, as well as of the frequency of horizontal transfer potential of ARG was evaluated in six commercial dairy farms in Ontario, Canada (Tran et al., 2021). Anaerobic digestion significantly decreased the viable coliform counts, the frequency of the horizontal transfer of ESBL genes as well as the abundance of sulphonamides, macrolides and beta-lactam resistance genes (Tran et al., 2021).

#### Composting techniques

Composting can reduce populations of ARB as well as ARG more effectively than other manure treatment processes, such as AD or simple manure stockpiling, but this depends on the type of manure and composting duration (Wang et al., 2015; Youngquist et al., 2016). In short-term cow manure stockpiles, the prevalence of antibiotic-resistant *E. coli* and *Enterococcus* spp. was not changed during 3 days (Walczak and Xu, 2011). In exchange, the composting of pig manure for 48 days led to a drastic decrease of ARB (*Acinetobacter* sp.*, Pseudomonas* sp.) by 4–7 log units in cultivable erythromycin-resistant and tetracycline-resistant bacteria (especially for *Acinetobacter* sp. strains) and of associated ARG (Wang et al., 2015). Hartmann and colleagues detected ESBL producing *E. coli* that had an identical rep-PCR pattern in animal fecal samples, composted manure and the environment of farms including cultivated and pasture fertilized soils, suggesting that ARB may persist in finished composts and facilitate their dissemination in fertilized soils (Hartmann et al., 2012).

Composting of the solid fraction of swine manure resulted in a reduction of different ARG concentrations, i.e.: *tet (Q), tet (W), tet (C), tet (G), tet (Z), tet (Y), tet (M), tet (W), tet (O), tet (T), erm (A), erm (C), erm (F), erm (T), erm (X), sul1, sul2, dfrA1, dfrA7, gyrA and parC* (Selvam et al., 2012; Wang et al., 2012). The broiler chicken litter was evaluated before and after composting for the abundance of 10 gene targets associated with antibiotic resistance or horizontal gene transfer (qPCR) and the results were correlated with the composition of the bacterial communities (16S rRNA gene amplicon sequencing) and the abundance of viable enteric bacteria (viable plate count; Subirats et al., 2020). Composting significantly reduced the abundance of enteric bacteria, including those carrying antibiotic resistance in litter from broiler chickens fed both with antibiotic supplemented diet and with antibiotic-free diet; the absolute abundance of all of the target genes decreased after composting except *sul1, intI1, incW* and *erm(F)* that remained stable (Subirats et al., 2020).

However, composting could lead to an apparently limited decrease in different representative ARG. For example, it has been shown that the chicken litter from broilers fed with bacitracin methylene disalicylate supplemented diet had an increased abundance of some ARGs, maintained after composting (Subirats et al., 2020). Le Devendec et al. (2016) indicated that, even after 6 weeks of composting or storage, resistance plasmids could still be transferred, suggesting that, in these conditions, composting may be insufficient to completely eliminate the risk of spreading AMR through chicken manure. Other studies have also shown that many ARGs persist after composting, with over 50 of these ARGs detectable, for example, in finished cattle manure composts (Qian et al., 2016). Co-composting of pig and chicken manure did not reduce the diversity of ARG, and a total of 19 ARG subtypes and two transposon genes were still persistent (Gao et al., 2019).

Several studies have indicated that temperature is a critical factor to limit the proliferation and activity of ARB and ARG (He et al., 2014; Wang et al., 2017). It has been shown that thermophilic composting has a higher efficiency for ARG removal than mesophilic composting (Sun et al., 2015; Qian et al., 2016). Pu et al. observed a decline in ARB, ARG and transposons abundance after aerobic co-composting of pig and chicken manures. However, the diversity and abundance of ARG was increased at the temperature-decreasing stage (55°C–25°C, 14–20 days), compared with the temperature-increasing stage (55°C–60°C, 3–14 days). Three genes conferring resistance to amphenicol macrolide-lincosamide-streptogramin B and vancomycin were highly enriched (101-fold, 420-fold, and 250-fold) at temperature decreasing stage (Pu et al., 2019).

## Conclusions and perspectives

The four major knowledge gaps related to the environmental dimensions of AMR proposed by Larsson et al. (2018), i.e., (Accinelli et al., 2007) the relative contributions of AMR from different sources (Agga et al., 2020) the role of the environment in the spread of AMR (Agga et al., 2022) the risk of human exposure to AMR in the environment, and (Alexy et al., 2004) the development of mitigation strategies, are all applicable to livestock production and manure as sources of antibiotic residues and AMR (Jutkina et al., 2018).

Fertilization with natural products is one of the main routes responsible for the introduction of antibiotic residues, ARG and zoonotic bacteria from animals to soil and, for the possible further dissemination into drinking water systems. Thus, proper handling, treatment and storage of manure prior to land application are key aspects to control the dissemination of AMR into the environment.

However, additional research is needed for understanding the fate of ARG during different types of manure treatment, such as anaerobic digestion and composting and to determine the optimal conditions for removal of antibiotic residues, ARB and ARG, allowing for the development of specific best practices for livestock manure treatment plants. It must also be determined if different manure treatments are really removing or just diluting resistance. Relevant criteria should be developed and/or modified according to research results for achieving a high-level efficiency in microorganisms removal during manure treatment and processing.

Another key intervention necessary to curb the further AMR emergence and spread and to maintain the efficiency of antibiotics is to limit their use, emphasizing the need for a prudent – cautious – use of antibiotics on farm animals to avoid unnecessary selective pressure (Hölzel et al., 2010). The proposed EU legislative framework, which will be enforced by 2022, bans the use of human reserve antibiotics in veterinary medicine and the use of unprescribed animal antimicrobials. Due to MDR organisms it is recommended that the “last resort” antibiotics and other clinically important antibiotic classes should be reserved for treatment of confirmed or suspected infections. An encouraging result of this measure is the decrease in the prevalence of colistin resistance genes in pig farm environments was reported in China after banning the use of colistin as an animal feed additive (Gao et al., 2019). Additionally, in order to safeguard the future use of antibiotics for treatment of bacterial infections, both in animals and humans, prudent use centered on correct diagnosis, correct choice and use of antimicrobials, along with appropriate susceptibility testing needs to be further strengthened (Silley and Stephan, 2017). On the other hand, it should be considered that antibiotic use in livestock is a prerequisite of animal welfare, consumer protection and cost efficiency of animal production. Further limiting the use of antibiotics on these farms might have effects on animal welfare and therefore might be difficult to implement (Wallace et al., 2018). However, a reduction of the consumption of quinolones and third-and fourth generation cephalosporins in veterinary medicine has been confirmed by the first joint report of the European Centre for Disease Prevention and Control (ECDC), the European Food Safety Authority (EFSA) (2018) and the European Medicines Agency (EMA) (2019) on antibiotics consumption and antibiotic resistance in humans and food-producing animals (Anon, 2015).

Further research should be also conducted to explore the potential dissemination routes of ARG among soil microorganisms and then to vegetables, to be able to take effective measures for controlling the persistence and dissemination of AMR in the vegetable production chain (Carballo et al., 2013). Additional research should follow to elucidate which microorganisms participate in the ARG transmission in a certain environment and quantify them.

The answers to these questions are pending on the characterization of land microbiome before and after manure application, assessing the risk of different raw and pre-treated manure types with regards to AR dissemination, as well as the maintenance and rate of pathogen transfer from grassland to animals. This is an essential component of agri-food research to fully assess the risk that manure land spreading has on the transfer of AR pathogens into the food chain and to humans.

Most imperative is that stakeholders help advise these research efforts so that scientific findings are more easily translated into practical on-farm management decisions and support to best equip the involved actors with the resources and tools needed to respond to the global AMR crisis.

## Author contributions

MC and LM: conceptualization. LM, MP, MJ, NM, CP, and PS: writing—original draft preparation. GG, PS, HS, ET, SG, PB, FB, and PK: writing—review and editing. MC: supervision. AR: funding acquisition. All authors contributed to the article and approved the submitted version.

## Funding

This research was funded by COFUND-JPI-EC-AMR-ARMIS, Antimicrobial Resistance Manure Intervention Strategies, grant number 40/2018; CNFIS-FDI-2022-0675, UEFISCDI - PN-III-P4-PCE2021-1797 and the Ministry of Research, Innovation and Digitalization through Program 1—Development of the national R&D system, Subprogram 1.2—Institutional performance—Financing projects for excellence in RDI, Contract no. 41 PFE/30.12.2021.

## Conflict of interest

The authors declare that the research was conducted in the absence of any commercial or financial relationships that could be construed as a potential conflict of interest.

## Publisher’s note

All claims expressed in this article are solely those of the authors and do not necessarily represent those of their affiliated organizations, or those of the publisher, the editors and the reviewers. Any product that may be evaluated in this article, or claim that may be made by its manufacturer, is not guaranteed or endorsed by the publisher.
